# Supplementary material for: “You need to dispose of them somewhere safe”: Covid-19, masks, and the pit latrine in Malawi and South Africa
Source: PLoS One. 2022 Feb 22;17(2):e0262741. doi: 10.1371/journal.pone.0262741 (PMC8863218; doi:10.1371/journal.pone.0262741)
Supplement: Attachment — Submitted filename: Review comments.docx [file pone.0262741.s001.docx]

Line 38. There should be a full stop after South Africa. The purpose of this study ….

Line 39 remove double space between “past years”

Line 45, behavior? There should be consistency in the English style. Refer to line 38.

Line 93, remove comma behind sanitation.

Line 94, put a comma after toilets and “the” before household.

Line 97, no space to ….

Line 104, remove “in”

Line 107, carry a stigma

Line 114, by the trash …

Line 115, remove “there”

Line 124-134 are not required in the introduction. This is the result of the investigation. Instead, the behavioural perception about waste disposal should be expanded in the paragraph.

Line 133, as it sheds light on how ….

Line 144, airport (…

Revise lines 235-237 for clarity.

Line 265, As such, masks were feared by ….

Line 269, shorten its life but….

Line 335, adaption? Consider replacing with adoption

Line 359, persona affects? Please revise

Line 396-399, starting from “Specifically …..” was mentioned in the introduction. It is not needed here. Revise it to avoid redundancy.

Line 403-404, Thus, for them,

Line 422, However, the …

Line 443, practices that ignore …. That acknowledge …

The introduction lacks information on the transmission and the period Covid-19 coronavirus could remain active on surfaces (masks) despite claims that face masks could be a channel to transmit coronavirus to children. Please, refer to

“An Overview of the Pathogenesis, Transmission, Diagnosis, and Management of Endemic Human Coronaviruses: A Reflection on the Past and Present Episodes and Possible Future Outbreaks - https://www.mdpi.com/2076-0817/10/9/1108/htm”

“An evidence review of face masks against COVID-19 - https://www.pnas.org/content/pnas/118/4/e2014564118.full.pdf” and other relevant articles

The manuscript did not have a discussion section but rather a lengthy conclusion. Please, revise this appropriately. Discussion of results should be clearly appreciated, and results discussed making reference to relevant published articles to support your results.
