# Supplementary material for: “You need to dispose of them somewhere safe”: Covid-19, masks, and the pit latrine in Malawi and South Africa
Source: PLoS One. 2022 Feb 22;17(2):e0262741. doi: 10.1371/journal.pone.0262741 (PMC8863218; doi:10.1371/journal.pone.0262741)
Supplement: Attachment — Submitted filename: Response to Reviewers.docx [file pone.0262741.s002.docx]

To the editor and reviewers,

We want to thank everyone for their time and feedback. We have engaged broadly with the comments and integrated them as thoroughly as possible. Please see a few specific responses to the reviewers’ comments below:

Response to Reviewer 1

| Comment | Response | Action |
| --- | --- | --- |
| However, ethical issues was not mentioned under the methodology as well as informed consent by the participant | Thank you for the comment. We have add information on informed consent. | See line 160-162 |

Response to Reviewer 2

| Comment | Response | Action |
| --- | --- | --- |
| Authors should include a paragraph summarising the transmission and of coronaviruses via face masks and other means of transmission of the virus from person to person. | We have added a few lines on transmission and masks; however, we have wanted to keep it concise. We referenced the recommended literature, thank you. | See lines 60-62 |
| Also, discussion and conclusion should be revised appropriately. | We pulled apart the conclusion into a standalone discussion and conclusion. | See text |
| Individual grammatical corrections | Thank you for the individual grammatical corrections. We have gone through and made corrections where necessary. |  |
