# Supplementary material for: “You need to dispose of them somewhere safe”: Covid-19, masks, and the pit latrine in Malawi and South Africa
Source: PLoS One. 2022 Feb 22;17(2):e0262741. doi: 10.1371/journal.pone.0262741 (PMC8863218; doi:10.1371/journal.pone.0262741)
Supplement: Attachment — Submitted filename: Reviewer 2 comments.docx [file pone.0262741.s003.docx]

The study “You Need to Dispose of them Somewhere Safe”: Covid-19, Masks, and the Pit Latrine in Malawi and South Africa” provided a background of individual perception on the potential risk of face mask and disposal in public space/or pit latrines in low-income communities in Durban and Malawi. The manuscript was significantly improved and I recommend it for publication after addressing the minor comments below

Line 90, faecal

Line 91, space to dig

Line 98, found in pits

Line 103, undesirable body? Consider undesirable body fluid

Line 109, and the removal of trash from …

Line 122, Likhubula lacks

Line 144, predominantly

Line 188, disposed of

Line 191, two-thirds

Line 225-227, revise for clarity

Line 251, disposed of in other ways

Line 262, how to use

Line 276, common-sense

Line 291, adopted

Line 348-350, occasionally tossing affects? Revise for clearity

Line 370, Moreover,

Line 393, Thus, for them, disposal

Line 396, disrupted during pandemic. So, for the few

Line 412, However, the pit ….

Line 369, other illnesses

Line 407, dumping where? Pit latrine? Kindly mention it here
